# Supplementary material for: DMRforPairs: identifying Differentially Methylated Regions between unique samples using array based methylation profiles
Source: BMC Bioinformatics. 2014 May 15;15:141. doi: 10.1186/1471-2105-15-141 (PMC4046028; doi:10.1186/1471-2105-15-141)
Supplement: Additional file 2 — DMRforPairs output for the comparison of A431-MCF7 and NA17018-NA17105. Please start from the HTML files in each folder. Available via the BMC Bioinformatics website. [file 1471-2105-15-141-S2.zip › 1394847754114233_MOESM2_ESM/NA17105M_NA17018F/figures/36891.pdf]

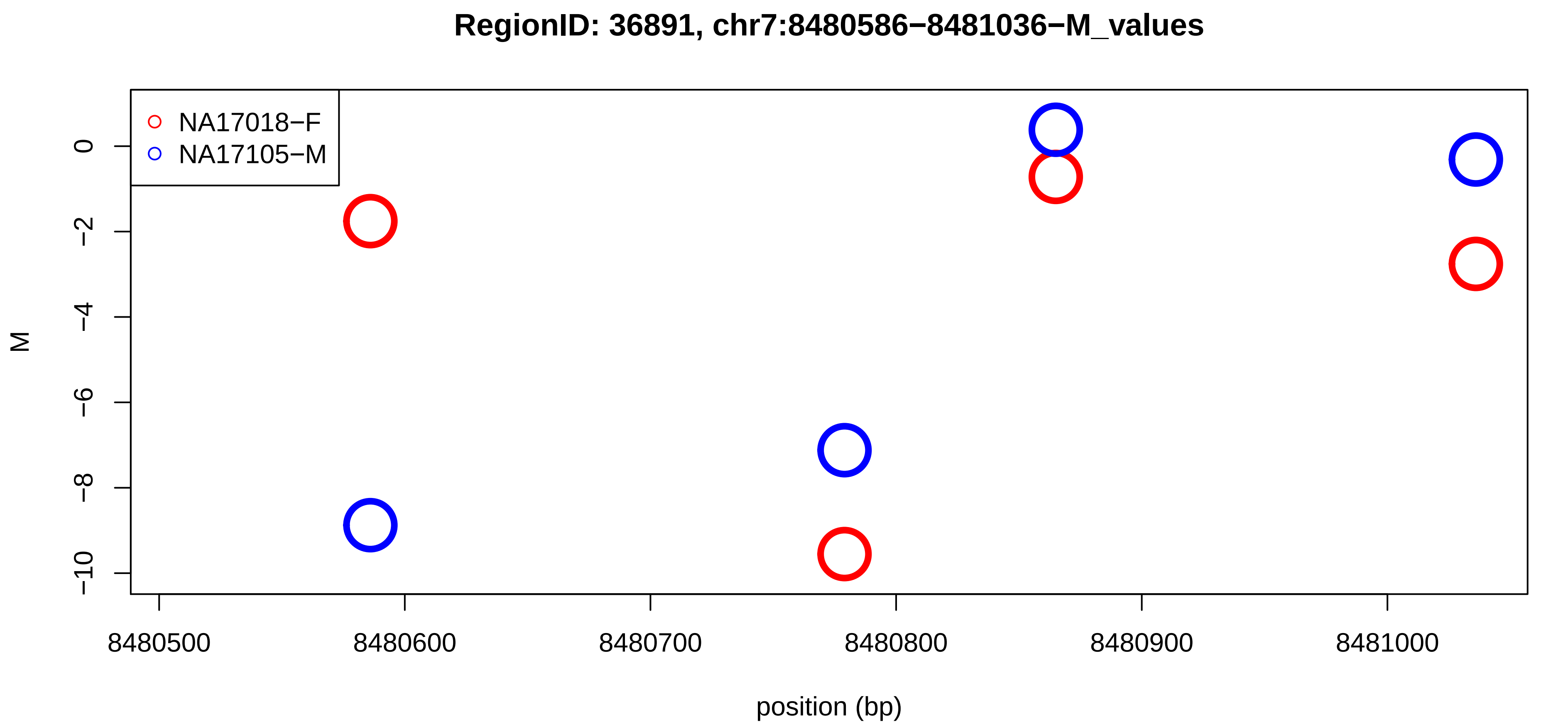
RegionID: 36891, chr7:8480586−8481036−M_values
NA17018−F
NA17105−M
M
0
−2
−4
−6
−8
−10
8480500
8480600
8480700
8480800
8480900
8481000
position (bp)

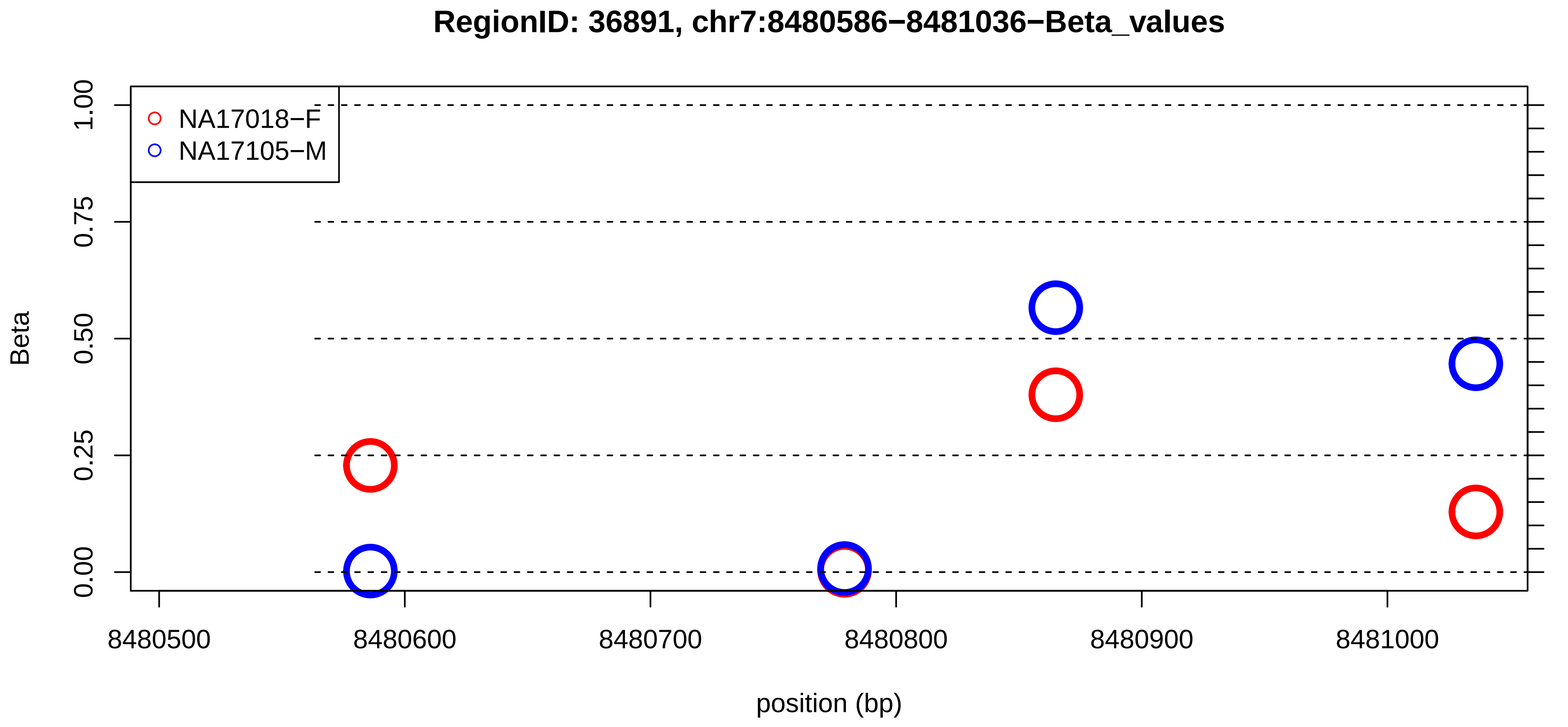
RegionID: 36891, chr7:8480586−8481036−Beta_values
NA17018−F
NA17105−M
Beta
1.00
0.75
0.50
0.25
0.00
8480500
8480600
8480700
8480800
8480900
8481000
position (bp)
